# Mechanism underlying polyvalent IgG-induced regulatory T cell activation and its clinical application: Anti-idiotypic regulatory T cell theory for immune tolerance

**DOI:** 10.3389/fimmu.2023.1242860

**Published:** 2023-11-29

**Authors:** Jefferson Russo Victor, Dong-Ho Nahm

**Affiliations:** ^1^ Laboratory of Medical Investigation LIM-56, Division of Dermatology, Medical School, University of Sao Paulo (USP), Sao Paulo, Brazil; ^2^ Post Graduation Program in Health Sciences, Santo Amaro University (UNISA), Sao Paulo, Brazil; ^3^ Department of Allergy and Clinical Immunology, Ajou University School of Medicine, Suwon, Republic of Korea

**Keywords:** immunoglobulins, T-lymphocytes, immunomodulation, regulatory T cell, atopic dermatitis, immune tolerance, allergic disease, autoimmune disease

## Abstract

The regulatory T (Treg) cells constitute a functionally defined subpopulation of T cells that modulate the immune system and maintain immune tolerance through suppression of the development of autoimmune responses to self-antigens and allergic reactions to external antigens. Reduction in the number or function of Treg cells has been suggested as a key immune abnormality underlying the development of autoimmune and allergic diseases. *In vitro* studies have demonstrated that purified polyvalent immunoglobulin G (IgG) from multiple healthy blood donors can exert immunomodulatory effects on Treg cells. Incubation of polyvalent human IgG with purified CD4^+^CD25^high^ T cells increased the intracellular expression of interleukin (IL)-10. Intravenous administration of polyvalent human IgG induced significant expansions of CD4^+^ Foxp3^+^ Treg cells and clinical improvements in patients with autoimmune diseases. In human clinical trials, intramuscular administration of autologous total IgG significantly increased the percentage of IL-10-producing CD4^+^ Treg cells in the peripheral blood of healthy subjects and provided significant clinical improvements in patients with atopic dermatitis. These results suggest a clinical usefulness of polyvalent IgG-induced activation of Treg cells in human subjects. This review proposes a new hypothesis for immune tolerance mechanism by integrating the pre-existing “idiotypic network theory” and “Treg cell theory” into an “anti-idiotypic Treg cell theory.” Based on this hypothesis, an “active anti-idiotypic therapy” for allergic and autoimmune diseases using autologous polyvalent IgG (as immunizing antigens) is suggested as follows: (1) Intramuscular or subcutaneous administration of autologous polyvalent IgG produces numerous immunogenic peptides derived from idiotypes of autologous IgG through processing of dendritic cells, and these peptides activate anti-idiotypic Treg cells in the same subject. (2) Activated anti-idiotypic Treg cells secrete IL-10 and suppress Th2 cell response to allergens and autoimmune T cell response to self-antigens. (3) These events can induce a long-term clinical improvements in patients with allergic and autoimmune diseases. Further studies are needed to evaluate the detailed molecular mechanism underlying polyvalent IgG-induced Treg cell activation and the clinical usefulness of this immunomodulatory therapy for autoimmune and allergic diseases.

## 1 Introduction

### 1.1 Immune tolerance and regulatory T cells

Immune tolerance is a state in which the immune system is unresponsive to foreign and self antigens that would otherwise result in a response (1). Immune tolerance is essential for maintaining immune homeostasis in healthy human subjects; defects in immune tolerance cause autoimmune and allergic diseases (1, 2).

Regulatory T (Treg) cells constitute a functionally defined subpopulation of T cells that modulate the immune system and have been suggested to play a central role in the maintenance of immune tolerance to self and foreign antigens through suppression of uncontrolled exaggerated autoimmune responses and allergic reactions that can be harmful to the host, thereby preventing the development of autoimmune and allergic diseases (1).

Treg cells are classified into natural Treg cells (nTreg cells) and induced Treg cells (iTreg cells) (3). nTreg cells arise from immature T cells in the thymus and express forkhead box P3 (Foxp3), CD4, and CD25 markers (4–6) and mediate peripheral immune tolerance through contact-dependent suppressor activity on other lymphocyte clones before they develop into full effector cells (7). iTreg cells develop after T cell maturation, do not express Foxp3, and can suppress other lymphocytes in peripheral tissues and lymph nodes but not through a contact-dependent mechanism; additionally, iTreg cells produce immunosuppressive cytokines (8). iTreg induction can be mediated through the exposure of naive CD4^+^ T cells to transforming growth factor-β (TGF-β), retinoic acid, and antigen presentation in peripheral tissues (3). In peripheral immune tolerance, repeated antigen exposure can also lead to the induction of iTregs (8). Among iTreg cells, the interleukin (IL)-10-producing CD4^+^ Treg cells (type 1 regulatory T cell: Tr1 cell) have been shown to play a key role in antigen-specific immune tolerance and can be induced through repeated administration of a specific antigen in humans (9–12). Foxp3^+^ Treg cells are primarily generated in the thymus (tTreg), but they can also be generated extrathymically at peripheral sites (pTreg) (13). Treg cells induce cytokine-dependent immune suppression through the secretion of IL-10 which suppresses Type 1 (Th1) and Type 2 T helper (Th2) cells (1, 2). Based on animal studies, decreased number and/or defective function (deficiency or dysfunction) of Treg cell have been suggested as a critical immune abnormality responsible for the development of autoimmune and allergic diseases (1, 2).

### 1.2 Polyvalent immunoglobulin G for immune regulation

Intravenous immunoglobulin G (IVIg), that is polyvalent IgG purified from the plasma pool of multiple healthy human blood donors, has been used to treat patients with primary immunodeficiency diseases associated with reduced immunoglobulin production (14, 15). Due to its immunomodulatory effects, IVIg has also been used to treat various autoimmune and allergic diseases (16, 17). Current evidence suggests that polyvalent IgG modulates the function of various immune cells, including dendritic cells (DCs), neutrophils, monocytes, macrophages, T cells, and B cells (18). Under *in vitro* experimental conditions, incubation of polyvalent human IgG with purified CD4^+^CD25^high^ T cells increased the expression of intracellular IL-10 in these cells (19). Activation of Treg cells seems to be the central mechanism responsible for the immunomodulatory and anti-inflammatory effects of polyvalent IgG (20–22).

The induction or activation of CD4^+^ Treg cells by IgG may involve diverse mechanisms considering the natural structure of these molecules. Some studies demonstrated that fragment crystallizable (Fc) regions of IgG obtained from IVIg formulations may interact with Fcγ receptors and thereby mediate part of the IVIg immunoregulatory effect (23, 24), while other report indicated that Fc fragments are not involved in this effect (25). Studies using recombinant IgG Fc fragments in murine models demonstrated a reduction in collagen-induced arthritis, idiopathic thrombocytopenic purpura, and myasthenia gravis (26–28), along with some beneficial effects in murine inflammatory neuropathy (29). The immunomodulatory effect of recombinant Fc fragments observed in the mouse model of collagen-induced arthritis could not be reproduced in macaques (28). In humans, the *in vivo* use of IVIg Fc fragments suggested a similar immunomodulatory effect of full IVIg in children with thrombocytopenic purpura (30); however, this effect could not be reproduced in *ex vivo* assays using human whole blood (31), and no other study could clarify the role of Fc fragments in human diseases (32).

The IgG fragments that can react with the antigen-binding sites (idiotypes) of pathogenic antibodies (IgG autoantibodies or IgE antibodies) in IVIg formulations have been suggested to mediate several aspects of immunomodulation through not only neutralization of pathogenic antibodies but also through other mechanisms, such as inhibition of complement activation and suppression of cytokine, in autoimmune and allergic diseases (33–35).

### 1.3 Idiotypic network theory

The “idiotypic network theory” that was initially proposed as a mechanism of immune tolerance by Jerne in 1974 suggests that the idiotypes of autologous immunoglobulins are immunogenic enough to induce immune responses (anti-idiotypic immune response) in the same host (36, 37). In this theory, the induction of an autoimmune anti-idiotypic response to the original idiotype plays a physiological role in the development of the immune tolerance state of the host. In this theory, immune tolerance is not a passive state of the immune system not responding to self and foreign antigens, but an active state of the immune system maintained through continuous low-grade physiological autoimmune responses to idiotypes of circulating autologous immunoglobulins in mammalian hosts preventing the development of exaggerated immune responses to self or foreign antigens (bacteria, virus, allergens) harmful to the host (36, 38, 39). Immune tolerance is a state of well-controlled responsiveness of the immune system to specific substances or tissues that would otherwise have the capacity to destroy these substances or tissues (36, 38, 39). Immune tolerance is induced via exposure to specific antigens through T cell receptors (TCRs) (1, 2). In the “idiotypic network theory”, idiotypes (variable regions with antigen specific binding capacity) of polyvalent autologous immunoglobulins and TCRs act as numerous endogenous antigens that can stimulate the immune system. These antigenic stimulations maintain numerous T cells and B cells with binding specificities to numerous antigens (both autoantigens and external antigens) and ultimately maintain an immune tolerance state (immune homeostasis).

This review focuses on mechanisms underlying the immunomodulation mediated by idiotypes of polyvalent IgG (including hypervariable regions of IgG) and not those mediated by the constant region of polyvalent IgG.

### 1.4 Anti-idiotypic regulatory T cell theory for immune tolerance

Unfortunately, knowledge regarding the scientific link between the pre-existing “Treg cell theory” and the “idiotypic network theory” for immune tolerance mechanisms is lacking. The mechanism underlying the cause-effect relationship between Treg cell activation and anti-idiotypic immune response has not been explained as yet. The disadvantages and weaknesses in the “idiotypic network theory” include the lack of experimental evidence regarding the actual mechanism in animals or humans through which immune tolerance is maintained and the absence of a human clinical trial demonstrating its clinical usefulness in treating autoimmune and allergic diseases. Rationale supporting the existence of “anti-idiotypic T cells” can be obtained from the “idiotypic network theory” itself. The “idiotypic network theory” suggests that the idiotypes of autologous immunoglobulins are immunogenic enough to induce immune responses (anti-idiotypic B cell and T cell immune response) in the same host (36, 37). Therefore, the pre-existence of anti-idiotypic T cells specifically recognizing peptides derived from idiotypes of autologous IgG is essential to maintain an immune tolerance state according to the “idiotypic network theory”. Thus, we propose a new hypothesis regarding the immune tolerance mechanism by integrating the “idiotypic network theory” and “Treg cell theory” into an “anti-idiotypic Treg cell theory” in this review.

T cells need antigenic stimulations for activation and survival (1, 3). What are the antigens stimulating Treg cells? This review proposes an answer that major antigens stimulating Treg cells include idiotypes (or its peptides processed by antigen-presenting cells, APCs) of autologous immunoglobulin.

## 2 Four hypothetical models for the mechanism underlying polyvalent IgG-induced activation of Treg cells

**Figure 1:**
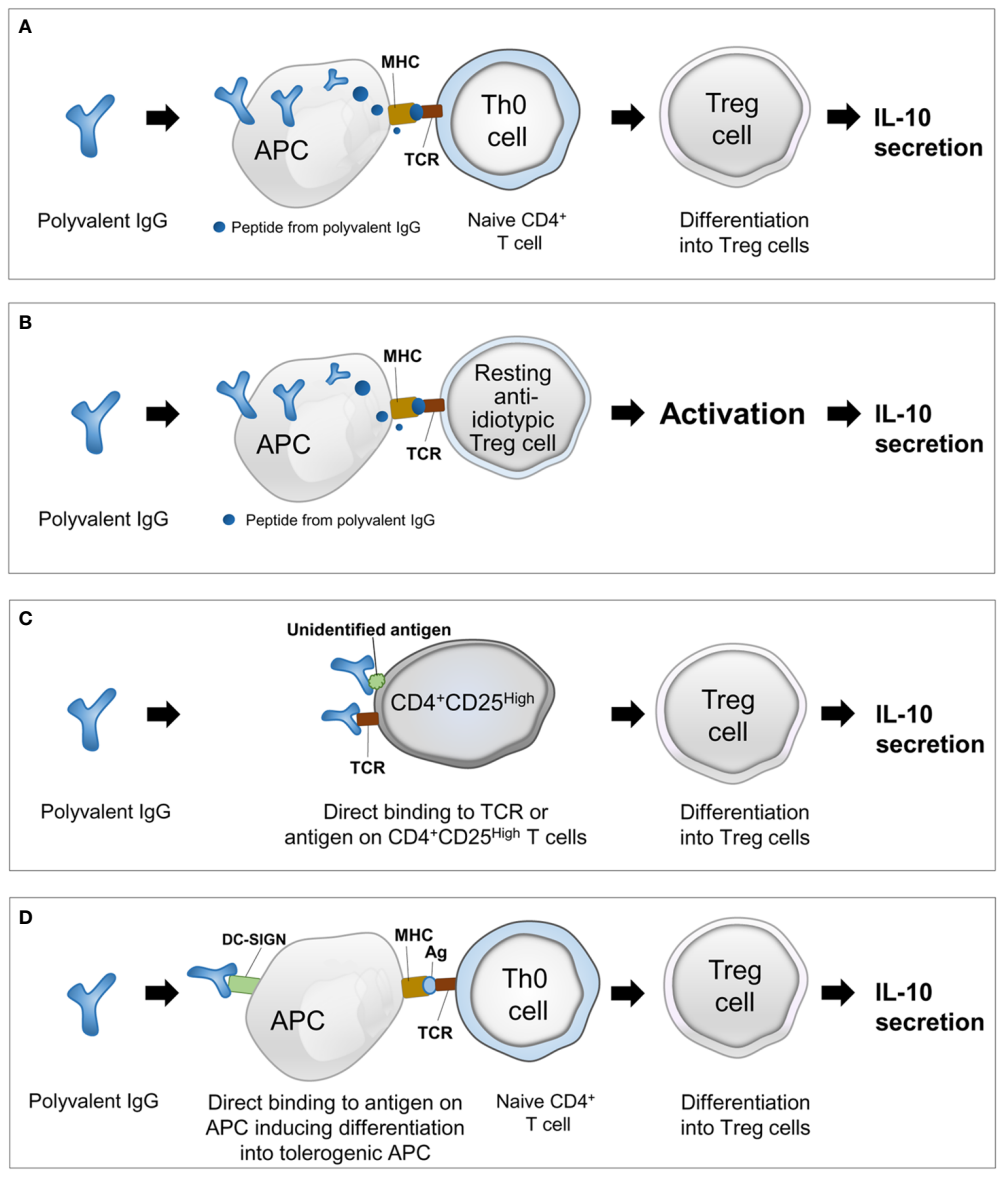
Four hypothetical models explaining the mechanism underlying polyvalent IgG-induced activation of regulatory T cells. **(A)** Presentation of peptides from polyvalent IgG by APCs and differentiation of naive T cells into Treg cells; **(B)** presentation of peptide idiotypes of polyvalent IgG by APCs and activation of pre-existing resting anti-idiotypic Treg cells; **(C)** activation of resting Treg cells by direct binding of polyvalent IgG to TCRs or unidentified antigens; and **(D)** direct binding of polyvalent IgG to conserved receptors or other unidentified antigens on APCs and induction of the tolerogenic phenotype of APCs facilitating differentiation of naive T cells into Treg cells. IgG, immunoglobulin G; Treg, regulatory T cell; IL-10, interleukin-10; TCR, T-cell receptor; APC, antigen-presenting cell; MHC, major histocompatibility complex; DC, dendritic cell; DC-SIGN, dendritic cell-specific intercellular adhesion molecule-3-grabbing nonintegrin.

This review proposes four hypothetical models for the mechanism underlying polyvalent IgG-induced activation of Treg cells on the basis of previous studies and our speculations (**Figure 1**).

[Model 1] Antigen presentation to naive T cells (Th0 cells): presentation of peptides from polyvalent IgG by APCs and differentiation of naive T cells into Treg cells (**Figure 1A**).

[Model 2] Antigen presentation to resting Treg cells: presentation of peptides from idiotypes of polyvalent IgG by APCs and activation of pre-existing resting anti-idiotypic Treg cells (**Figure 1B**).

[Model 3] Direct binding to antigens on T cells: activation of resting Treg cells by direct binding of polyvalent IgG to TCRs or unidentified antigens (**Figure 1C**).

[Model 4] Direct binding to antigens on APCs: direct binding of polyvalent IgG to conserved receptors or other unidentified antigens on APCs and induction of the tolerogenic phenotype of APCs facilitating differentiation of naive T cells into Treg cells (**Figure 1D**).

However, these four hypothetical models should be validated through future experiments. This review describes the limitations (unanswered questions) of these hypotheses to promote further studies on this topic by colleagues (**Table 1**).

**Table 1:** Six general questions on the mechanism of immune modulation and activation of regulatory T cells induced by polyvalent IgG.

Questions:
*1) How can variable portions of polyvalent IgG (idiotopes or idiotype) induce activation of Treg cells?* *2) Can anti-idiotypic T cells act as Treg cells?* *3) What is the antigen-specificity of Treg cells activated by polyvalent IgG?* *4) Which subpopulation of Treg cells is activated by polyvalent IgG (CD4^+^Foxp3^+^ Treg cells and/or CD4^+^Foxp3^-^IL-10^+^ Treg cells)* *5) Is the anti-idiotypic Treg cell-mediated immunomodulation a physiological mechanism working in healthy human subjects to maintain immune tolerance?* 6) *Could immunization with autologous polyvalent IgG trigger the activation of anti-idiotypic Treg cells, leading to a long-term clinical remission in patients with autoimmune and allergic diseases through the induction of immune tolerance?*

IgG, immunoglobulin G; Treg cell, regulatory T cell; Foxp3, forkhead box P3; IL, interleukin.

### 2.1 Hypothetical model 1 and 2: activation of Treg cells by peptides from polyvalent IgG processed by APCs (main hypotheses of authors)

APCs, including DCs, take up antigens in peripheral tissues, process them into peptides through proteolysis, and load these peptides on major histocompatibility complex (MHC) class I and II molecules (40). This review suggests that APCs, including DCs, can phagocytose polyvalent IgGs and present the peptides to naive T cells, after which the naive T cells differentiate into Treg cells [Model 1], or polyvalent IgG derived peptides activate resting anti-idiotypic Treg cells to produce IL-10 [Model 2] (**Figures 1A, B**). The following are past studies supporting these two hypothetical models.

In an abortion-prone mouse model, peptides produced by APCs from processing of the constant region of polyvalent IgG with a high affinity to MHC class II molecules (that can activate Treg cells and are termed as Tregitopes) can reduce the fetal death rate and increase IL-10 producing CD4^+^CD25^+^Foxp3^+^ Treg cells (41). In a mouse model of ovalbumin (OVA)-induced asthma, intraperitoneal administration of IVIg from healthy human blood donors could induce antigen-specific peripherally generated Foxp3^+^ Treg cells even after selective depletion of pre-existing CD4^+^CD25^+^Foxp3^+^ Treg cells and inhibit Th2 responses and airway inflammation (42). Subsequently, the same research group demonstrated that the inhibitory effect of Tregitopes could promote tolerance by activating Treg activity in a mouse model of OVA-induced asthma (43). These results suggested that Tregitopes could be active components mediating IVIg-induced anti-inflammatory effect (Model 1, **Figure 1A**).

In a murine model of OVA-immunization to induce OVA-specific IgG antibodies, subcutaneous injections of IVIg induced the activation and expansion of B and CD4^+^ T cells in the spleen and draining lymph nodes, resulted in a reduction in OVA-specific antibody production, and induced anti-IVIg IgG antibodies that mainly recognized the F(ab’)_2_ fragments of IVIg (44). These immunomodulatory effects could not be induced after subcutaneous injections of two human IgG monoclonal antibodies that share the constant region of IgG but not their idiotypes (44). These observations suggested that the immune responses to idiotypes of IVIg with enormous sequence diversity might produce immunomodulatory effects suppressing the production of anti-OVA IgG antibodies (44). However, in this mouse model, there was no evidence of the activation of Treg cells induced through the subcutaneous injections of IVIg (44). Investigation of the human leukocyte antigen (HLA) class II peptide repertoire from IVIg-loaded human DCs via MHC-associated peptide proteomics (MAPPs) revealed that numerous peptides derived from the hypervariable region of IgG were strongly presented (45). Surprisingly, Tregitopes derived from the constant region of IgG failed to suppress effector immune responses against a specific antigen in both human peripheral blood mononuclear cells and mouse immune cells (45). These results suggested that the immunomodulatory effects of polyvalent IgG were originated from the hypervariable region (idiotype) of IgG and not from the constant region of IgG (Tregiotopes) (Model 2, **Figure 1B**).

In humans, an author of this review (Nahm DH) reported that the intramuscular administration of autologous total IgG increased the percentage of IL-10-producing CD4^+^ T cells in the peripheral blood in 13 healthy human subjects (46). However, this report could not provide a detailed molecular mechanism underlying the autologous polyvalent IgG-mediated immunomodulation or the subpopulation of Treg cells activated by autologous polyvalent IgG.

This review provides a summary of results (subpopulation of Treg cells and mechanism) on the polyvalent IgG-induced activation of Treg cells from mouse studies (21, 42, 44, 47–51) (**Table 2**). Further studies are needed to evaluate the characteristics of polyvalent IgG-derived peptides activating Treg cells and subpopulations of Treg cells mediating polyvalent IgG-induced immunomodulation.

**Table 2:** Effect of polyvalent IgG-induced regulatory T cell activation in mouse model.

Induced disease	lineage	IgG type	Administration dose (route)	Evaluation timing	Specimen source	Measurement of Treg cell	Immunological effect (or mechanism)	Ref
Experimental autoimmune encephalomyelitis (EAE)	Mouse C57BL/6J female	Human IVIg	0.8 g/kg (IP)	Day 21	Spleen	↑ CD4^+^CD25^+^Foxp3^+^	Protection by IVIg was associated with an increase in CD4^+^CD25^+^Foxp3^+^ Treg cell numbers and function. IVIg failed to protect against EAE in Treg cell-depleted mice. IVIg had a direct effect on the proliferation of natural Treg cells.	(21)
OVA-induced airway hyperresponsiveness (AHR)	Mouse C57BL/6 male and female	Human IVIg	2 g/kg (day 28) (IP)	Day 32	Lung	↑ CD4^+^CD25^high^ Foxp3^+^	IVIg generated a *de novo* population of peripheral Treg (pTreg) cells in the absence of endogenous Treg cells. IVIg-generated pTreg cells were sufficient for inhibiting OVA-induced AHR. Adoptive transfer of purified IVIg-generated pTreg prior to antigen challenge effectively prevented airway inflammation and AHR in an antigen-specific manner.	(42)
OVA-induced IgE response	Mouse C57Bl/6NCrl female	Human IVIg	1mg, 10mg, 20mg, 50mg (day 0, day7, and day 14) (SC)	Day 15	Spleen LN	No difference of percentage CD4^+^CD25^+^ Foxp3^+^ between IVIg treatment group and control group	IVIg induced the activation and expansion of B and CD4^+^ T cells, reduced development of OVA-specific antibody, and induced anti-IVIg IgG antibodies that mainly recognizing the F(ab’)_2_ fragments of IVIg.	(44)
OVA-induced IgE response	Mouse BALB/c male and female	OVA-immunized mouse IgG	600 ug/per pregnant mice at prenatal period (divided in 3 days at days 10,15 and 20) (IV)	Day 20	Spleen	↑ L-10-producing CD4^+^	Passive transfer of OVA-immunized IgG to pregnant mice increased the frequency of IL-10-producing T cells in offspring and prevented neonatal OVA-sensitization.	(47)
Collagen-induced arthritis (CIA)	Mouse DBA1/J male	Human IVIg	25 mg/mouse at day 7 (IV)	Week 15	Spleen	↑ CD4^+^CD25^+^ Foxp3^+ ^(FACS) ↑ CD4^+^CD25^+^ Foxp3^+^IL-10^+^ (confocal microscopy)	IVIg increased the number of Foxp3^+^ Treg cells. The therapeutic effects of IVIg are dependent on IL-10. The treatment effects of IVIG on arthritis were lost in IL-10-knockout mice.	(48)
Herpes simplex virus (HSV) induced fatal encephalitis	Mouse 129S6 male	Human IVIg	3.75 mg/mouse (IP)	Day 1 after HSV infection	Spleen	↑ CD4^+^CD25^+^Foxp3^+^	IVIg induced secretion of IL-10 from Treg cells and it produced anti-inflammatory effects that protect against fatal HSV encephalitis.	(49)
OVA-induced airway hyperresponsiveness (AHR)	Mouse C57BL/6	Human IVIg	2 g/kg 24hr before antigen challenge (day 28)(IP)	After 5 days of OVA or sham challenge	Spleen LN Lung	↑ CD4^+^Foxp3^+^	IVIg induced antigen-specific Foxp3^+^ Treg cells from non-Treg cell precursors. Induction of Treg cells was mediated by tolerogenic dendritic cells.	(50)
Experimental autoimmune encephalomyelitis (EAE)	Mouse C57BL/6J female	Human IVIg	0.8 g/kg/daily from day 0 to day 18 (IP)	Day 9	Spleen	↑ CD4^+^Foxp3^+^	IVIg inhibits the differentiation of naive CD4 T cells into EAE Th1/17 cells and induced an expansion of Foxp3^+^ Treg cells. F(ab’)_2_ fragments retained this function of IVIg.	(51)

IgG, immunoglobulin G; IgE, immunoglobulin E; Treg cell, regulatory T cell; IVIg, purified polyvalent IgG from the plasma pool of multiple healthy human blood donors; Foxp3, forkhead box P3; OVA, ovalbumin; SC, subcutaneous; IP, Intraperitoneal; IV, intravenous; LN, lymph node; IL, interleukin; FC, fragment crystallizable.

### 2.2 Hypothetical model 3 and 4: activation of Treg cells by direct binding of polyvalent IgG to surface receptors on T cells or APCs

Addition of polyvalent human IgG to purified CD4^+^CD25^high^ T cells from healthy human subjects increased intracellular expression of IL-10 in CD4^+^CD25^high^ T cells under experimental cell culture conditions; this observation indicated a direct activation of Treg cells by polyvalent IgG (20). To explain the mechanism underlying polyvalent IgG induced direct activation of T cells, the “hook without bait theory” has been proposed by an author of this review (Victor JR) (52). In this theory, direct binding of idiotype of polyvalent IgG to the TCRs or unidentified antigens on the surface of CD4^+^ cells can induce differentiation of Treg cells from naive T cells or activate pre-existing Treg cells to secrete IL-10 (**Figure 1C**). The direct interaction between polyvalent IgG and antigens on the cell membrane of CD4^+^ thymic T cells induced cytokine production from CD4^+^ thymic T cells (53). However, this study did not evaluate the activation of Treg cells by direct binding of polyvalent IgG to antigens on the cell membrane of T cells. In a co-culture model of human DCs and CD4^+^ T cells as well as in a mouse model of experimental autoimmune encephalomyelitis, IVIg was seen to increases Treg cells through the binding of F(ab′)_2_ fragments of IVIg with dendritic cell-specific intercellular adhesion molecule-3-grabbing nonintegrin (DC-SIGN) on DCs (54) (**Figure 1D**). A similar approach demonstrated that polyvalent IgG can specifically interact with murine DC immunoreceptors, favoring the induction of Treg cells (55). Another possibility is the direct binding of polyvalent IgG to an unidentified antigen (or conserved receptor) on the surface of APCs, and this interaction induces the changes of APCs into tolerogenic APCs, promoting the differentiation of naive CD4^+^ T cells into Treg cells and secretion of IL-10 (**Figure 1D**).

However, further studies are needed to validate these four hypothetical models of polyvalent IgG-induced Treg cell activation and the subpopulations of Treg cells (CD4^+^Foxp3^+^ T cells and/or Tr1 cells) involved in these processes.

## 3 Evidence supporting activation of Treg cells by polyvalent IgG in clinical trials

### 3.1 Evidence supporting activation of Treg cells upon intravenous administration of IVIg in human clinical trials

IVIg has been used as a replacement or immunomodulatory therapy in primary immunodeficiency, secondary immunodeficiency, hematological diseases, neuroimmunological diseases, rheumatic diseases, dermatological diseases, and other conditions including miscarriages (56). Clinical studies on IVIg therapy in primary immunodeficiency patients (57), the treatment of autoimmune rheumatic disease (58), Guillain-Barré syndrome (59), eosinophilic granulomatosis (60), Kawasaki disease (61), recurrent miscarriage (62), and repeated implantation failure (63) showed that IVIg therapy increased the percentage of Treg cells (mainly CD4^+^ Foxp3^+^ Treg cells) in the peripheral blood (**Table 3**). In a clinical trial, IVIg therapy in patients with common variable immunodeficiency significantly increased the number of peripheral Treg cells (expressing CD4, CD25, and low levels of CD127) and plasma levels of IL-10 within 30 minutes after the infusion of IVIg, suggesting a highly efficient interaction between polyvalent IgG and Treg cells (64). This review provides a summary of study results (subpopulation of Treg cells and mechanism) on the polyvalent IgG-induced activation of Treg cells in human clinical trials (**Table 3**) (46, 57–65).

**Table 3:** Effect of polyvalent IgG-induced regulatory T cell in human clinical trials.

Treatment-target diseases	Study design	IgG type	Administration dose (route)	Evaluation timing	Measurementof Treg cell	Immunological effect (or mechanism)	Ref
Primary immunodeficiency	Prospective uncontrolled	IVIg	0.36-0.72 g/Kg for 1 day (IV)	Pre-infusion *vs.* 7 days after infusion	↑ CD3^+^CD4^+^CD25^High^ Foxp3^+^	Anti-inflammatory effects of IVIg inducing expansion of Treg cells was noticed as early as day 7.	(57)
Autoimmune rheumatic disease	Prospective uncontrolled	IVIg	2 g/Kg (IV)	Pre-infusion *vs.* 72 to 96 hour after last infusion	↑ CD4^+^CD25^high^	Anti-inflammatory effects of IVIg with enhancement of Treg cells were observed.	(58)
Guillain-Barré syndrome	Prospective controlled	IVIg	0.4 g/Kg/daily for 4 days (IV)	Pre-infusion (0 week) *vs.* 1 week after infusion	↑ CD4^+^Foxp3^+^	IVIg reduced the frequency of Th1 and Th17 cells and expanded Treg cells. IVIg-expanded Treg cells exhibited T cell suppressive function.	(59)
Eosinophilic Granulomatosis	Prospective controlled	IVIg	0.4 g/kg/daily for 5 days (IV)	IVIg *vs.* without IVIg (3, 6, 12 and 24 months after infusion)	↑ CD25^+^ among CD4^+^	The frequency of CD25^+^ cells among CD4^+^ T cells increased significantly after IVIg treatment.	(60)
Kawasaki disease	Prospective controlled	IVIg	2 g/kg (IV)	Pre-infusion *vs.*48 hour after infusion	↑ CD4^+^CD25^+^ Foxp3^+^	Percentage of CD4^+^CD25^+^Foxp3^+^ cells were significantly increased at 48 hour after IVIg.	(61)
Recurrent miscarriage	Prospective controlled	IVIg	0.4 g/kg every 4 weeks during 32 weeks of gestation (IV)	At the diagnosis of first positive pregnancy *vs.* at week 32 (last infusion of IVIg)	↑ CD4^+^CD25^+^ CD127^−^	IVIg induced a significant increase of Treg cells.	(62)
Repeated implantation failure	Prospective controlled	IVIg	0.4 g/kg (2 days before embryo transfer) (IV)	2 days before embryo transfer *vs.* 15 days after embryo transfer	↑ CD4^+^ Foxp3^+^ CD127^−/low^	IVIg induced a significant increase of Treg cells.	(63)
Common variable immunodeficiency	Prospective controlled	IVIg	0.3 g/kg by slow 2- hour (IV)	30 minutes before infusion *vs.*30 minutes after infusion	↑ CD4^+^ ↑ CD4^+^CD25^+^ CD127^−/low^	IVIg increased percentage of Treg cells as early as at 30 minutes after IVIg infusion.	(64)
Primary immune thrombocytopenia	Prospective controlled	IVIg	1 g/kg/day for consecutive 2 days (IV)	·Pre-infusion *vs.*2 days after infusion ·Pre-infusion *vs.* week 4 after infusion	↑ CD4^+^CD25^+^Foxp3^+^	IVIg induced expansion of Treg cells at day 2 and week 4 after infusion.	(65)
Healthy human subjects	Prospective uncontrolled	Autologous total IgG	0.4 g (8 injection of 50 mg per 4 weeks) (IM)	Pre-injection (0 week) *vs.* weeks 4, 8, and 12	↑ IL-10-producing CD4^+^ ↑ IFN-γ-producing CD3^+^	Intramuscular injection of autologous total IgG increased IL-10-producing CD4^+^ T cells at weeks 4, 8, and 12 compared to baseline (0 week).	(46)
Atopic dermatitis	RCT	Autologous total IgG	0.4 g (8 injection of 50 mg per 7 weeks) (IM)	Pre-injection (0 week) *vs.* week 16	↑ IL-10-producing CD4^+^	Intramuscular injection of autologous total IgG increased Treg cells at week 16 compared to baseline (0 week).	(66)

IgG, immunoglobulin G; Treg cell, regulatory T cell; IVIg, purified polyvalent IgG from the plasma pool of multiple healthy human blood donors; Foxp3, forkhead box P3; IV, intravenous; IM, intramuscular; IL, interleukin; IFN, interferon; RCT, randomized clinical trial.

### 3.2 Clinical application of “active anti-idiotypic therapy” with polyvalent IgG: lessons obtained from clinical trials on intramuscular injection of autologous total IgG in patients with atopic dermatitis

An author (Nahm DH) of this review has tried to develop a new anti-idiotypic therapy through intramuscular injection of autologous total IgG purified from autologous blood via affinity-chromatography using protein A bead in patients with atopic dermatitis (AD) and healthy human subjects (46, 66–70). From clinical trials in patients with AD and healthy human subjects, the novel concept of an “active anti-idiotypic therapy” was developed as described in the following sections.

#### 3.2.1 Current knowledge on pathogenesis and treatment of atopic dermatitis

AD is an allergic skin disorder characterized by chronic allergic inflammation of the skin with clinical manifestations including itching, dry skin, exudation, and frequently associated with a personal or familial history of allergic diseases (2, 71, 72).

In patients with AD, allergic inflammation of the skin is characterized by the uptake of allergens by APCs that digest allergens and present peptides of the allergen to allergen-specific CD4^+^ T cells, which secrete Th2 cytokines (IL-4, IL-5, and IL-13) (73). Upon re-exposure, allergens activate Th2 cells to secrete Th2 cytokines, and allergens also bind to IgE antibodies on the mast cells and induce mast cell degranulation and release of chemical mediators, including histamine and other chemo-attractants. These cytokines and chemical mediators induce chronic allergic inflammation (Th2 inflammation) of the skin and clinical symptoms of AD (itching and eczema) (73).

Current topical and systemic immunosuppressants (corticosteroids, cyclosporine, tacrolimus, and methotrexate) nonspecifically suppress inflammatory immune cells and have limited clinical efficacy in patients with moderate-to-severe AD (2). Recently developed monoclonal antibodies to IL-4 receptor alpha or Janus kinase inhibitors suppressing Th2 cell-mediated inflammation demonstrated clinical improvements in a significant number of patients with moderate-to-severe AD (74–77). However, the clinical efficacies of current medical therapies for AD are transient and remain incomplete (71, 78). Therefore, developing a new therapeutic modality modifying the long-term clinical course of AD is needed.

#### 3.2.2 Critical role of decreased number and/or function of Treg cells in the pathogenesis of atopic dermatitis

While Th2 cells may promote allergic inflammation, Treg cells can downregulate Th2 cell function and suppress allergic inflammations. T cell tolerance mediated by Treg cells is the key mechanism underlying immune tolerance in a healthy immune response to self and non-infectious foreign antigens. Immune dysfunction characterized by decreased Treg cell function (reflected by the decreased number and/or function of Treg cells) (79, 80) and excessive activation of Th2 cells producing IL-4 and IL-13 plays a key role in the pathogenesis of AD (2).

Therefore, immunomodulatory therapies to restore Treg cell number and/or function and Th1/Th2-cell balance might be a reasonable approach to induce a long-term treatment-free clinical remission in patients with AD (2, 81).

#### 3.2.3 Immunomodulatory therapies that induced a long-term treatment-free clinical remission in patients with atopic dermatitis

##### 3.2.3.1 Allergen immunotherapy

Allergen immunotherapy is a treatment that involves the repeated administration of sensitized allergens either subcutaneously or sublingually to induce allergen-specific immune tolerance in patients with allergic diseases (82, 83). Allergen immunotherapy is clinically beneficial in AD patients sensitized to house dust mites in a meta-analysis of multiple randomized clinical trials (84, 85). Clinical observational studies reported long-term treatment-free clinical remissions in patients with AD after allergen immunotherapy (86–88). Allergen immunotherapy activates allergen-specific Treg cells to downregulate Th2 cell- and IgE-mediated allergic inflammation. Treg cells are generated during allergen immunotherapy, secrete IL-10, and induce allergen-specific B cells to produce IgG4 antibodies (89). These mechanisms induce tolerance to antigens that reduce allergic symptoms. Induction of peripheral T cell tolerance by Treg cells is the key mechanism involved in allergen immunotherapy (90). Activated Treg cells release IL-10 and suppress Th2 cell-mediated allergic inflammation. However, there are still controversies regarding the main subpopulation of Treg cells mediating therapeutic effects involved in allergen immunotherapy (CD4^+^Foxp3^+^ Treg cells and/or CD4^+^Foxp3^-^IL-10^+^ Treg cells) (91).

##### 3.2.3.2 Natural remission of disease in children with atopic dermatitis

More than 70% of children with AD experience natural remission of their disease before puberty (92); although the mechanism of natural remission of AD in children has not been determined yet, induction of immune tolerance due to activation of Treg cells has been suggested as a mechanism (2). Induction of immune tolerance mimicking the immunological mechanism responsible for the natural clinical remission of AD in children could be an ideal way to achieve clinical remission in patients with AD (2).

##### 3.2.3.3 Intramuscular administration of autologous polyvalent IgG for induction of anti-idiotype immune modulation

An author of this review (Nahm DH) hypothesized that immunizing the human subjects with autologous immunoglobulin might induce Treg cell response with systemic immunomodulatory effects to prevent or treat AD. To evaluate this hypothesis, clinical trials were performed to evaluate the clinical efficacy and immunomodulatory effect of intramuscular administration of autologous polyvalent IgG (total IgG) purified from autologous blood using Protein A bead in patients with AD and healthy human subjects (46, 66–70).

In a randomized controlled trial including 51 patients with AD, 8 weekly intramuscular injections of autologous total IgG (50 mg) for 7 weeks resulted in significant clinical improvements and increased serum levels of IL-10 and interferon (IFN)-γ at week 16 (66). In this study, the percentages of IL-10- and IFN-γ-producing cells in the peripheral blood CD4^+^ T cells were increased after the intramuscular administration of autologous total IgG in patients with AD (66). These results suggested that intramuscular administration of low doses of autologous total IgG can activate Treg cells and Th1 cells producing IFN-γ and lead to systemic immunomodulatory effects in patients with AD (66).

In a previous clinical study, a long-term clinical improvement for more than nine months was observed in 2 of 3 patients with severe AD who received 8 intramuscular injections of autologous total IgG 50 mg (total 400 mg) for 4 weeks and were followed up for two years (69). In this study, two of three patients showed clinical improvement for more than 36 weeks after treatment with maximum decreases in clinical severity score greater than 80% from baseline. These two patients showed a long-term (more than 36 weeks) decrease in serum total IgE concentration and peripheral blood eosinophil count with maximum decreases in those values greater than 70% from baseline. No significant side effect was observed during the two years of follow-up period in all three patients (69). This observation suggests a potential of intramuscular administration of autologous total IgG in modifying the long-term disease course of AD.

Changes in T cells before and after intramuscular administration of autologous total IgG were evaluated in 13 healthy human subjects (46). Intramuscular administration of autologous total IgG (8 injections of autologous IgG 50 mg per 4 weeks; total 400 mg IgG) significantly increased the percentage of IL-10-producing CD4^+^ T cells in peripheral blood CD4^+^ T cells and the percentage of IFN-γ-producing CD3^+^ T cells in peripheral blood CD3^+^ T cells in healthy human subjects (46) (**Table 3**). These results suggest that intramuscular administration of autologous total IgG can activate Treg cells and IFN-γ-producing T cells in healthy subjects and that it could be a safe and effective method to activate Treg cells in human subjects (46). The significant limitations of these clinical studies are the lack of knowledge on the detailed molecular mechanism underlying Treg cell activation induced by autologous total IgG and the subpopulation of Treg cells (CD4^+^Foxp3^+^ Treg cells and/or CD4^+^Foxp3^-^IL-10^+^ Treg cells) activated by autologous total IgG.

An author of this review (Nahm DH) speculates that the intramuscular administration of autologous total IgG activates pre-existing anti-idiotypic Treg cells that can specifically recognize peptides derived from IgG idiotypes by their TCRs and produce IL-10, based on the observations in healthy human subjects after intramuscular injection of autologous total IgG (46). However, a direct experimental demonstration of the existence or development of anti-idiotypic Treg cells has not been provided yet. Therefore, an author (Nahm DH) of this review proposes that “anti-idiotypic Treg cell” is a missing scientific link that can integrate both “idiotypic network theory” and “Treg cell theory” to explain the mechanism underlying the maintenance of an immune tolerance state (**Tables 1**, **4**).

**Table 4:** Ten future research topics on the detailed mechanism underlying polyvalent IgG-induced activation of regulatory T cells and its clinical application.

Questions for future research topics
*1) Which subpopulation of Treg cells responsible for the immunomodulatory effect of polyvalent IgG? (Tr1 cells or Foxp3^+^ Treg cells)?* *2) Can the peptides from idiotypes of the IgG processed by antigen-presenting cells induce a differentiation of naive CD4^+^ T cells into Treg cells or an activation of pre-existing resting anti-idiotypic Treg cells?* *3) Can the direct binding of polyvalent IgG to T cell receptors on the surface of Treg or antigens in the antigen-presenting cells induce activation of Treg cells?* *4) Can intramuscular or subcutaneous injection of autologous polyvalent IgG induce activation of Treg cells specific to peptides derived from idiotypes of autologous IgG (anti-idiotypic Treg cells)?* *5) Can the existence of anti-idiotypic Treg cell be demonstrated by experiments in cell culture condition or animal models?* *6) Can intramuscular or subcutaneous injection of autologous polyvalent IgG increase the production of anti-idiotypic antibodies?* *7) Is there any difference in the immunomodulatory effects and clinical efficacy between intravenous injection of polyvalent IgG from healthy blood donors and intramuscular or subcutaneous injection of autologous polyvalent IgG in patients with allergic or autoimmune diseases?* *8) Is there a difference in immunomodulatory mechanism induced by intravenous injection of polyvalent IgG from healthy blood donors or intramuscular or subcutaneous injection of autologous polyvalent IgG?* *9) Can intramuscular or subcutaneous injection of autologous polyvalent IgG induce long-term clinical improvements in patients with allergic and autoimmune diseases more efficiently compared with intravenous injection of polyvalent IgG from healthy blood donors?* *10) Are the concepts of passive anti-idiotype therapy for the explanation of the therapeutic mechanism of intravenous injection of polyvalent IgG from healthy blood donors (supplementing natural anti-idiotypic antibodies to neutralize pathogenic antibodies) and active anti-idiotypic therapy for the induction of anti-idiotypic Treg cell and B cell responses by intramuscular or subcutaneous injection of autologous polyvalent IgG correct?*

IgG, immunoglobulin G; Treg cell, regulatory T cell; Tr1 cell, type 1 regulatory T cell; nTreg cell, natural regulatory T cell; Foxp3, forkhead box P3.

##### 3.2.3.4 Comparison of efficacy and mechanism of intravenous injection of heterologous polyvalent IgG and intramuscular injection of autologous total IgG in patients with atopic dermatitis

Expansion of CD4^+^ Foxp3^+^ Treg cells in the peripheral blood has been reported in patients with autoimmune diseases after intravenous administration of high-dose (1~2 g/Kg of body weight) heterologous IVIg (60, 65, 93). Continuous regular intravenous infusion of a high dose of polyvalent human IgG is needed to achieve a long-term clinical improvement in patients with autoimmune diseases (60, 91, 93). This requirement of continuous treatment has been attributed to the low concentration and low affinity of natural anti-idiotype antibodies to pathogenic autoantibodies contained in IVIg (17). Clinical trials on high-dose intravenous polyvalent IgG therapy (1~2 g/Kg of body weight per patient) in adult patients with severe AD showed no significant clinical benefit (93). However, intramuscularly administered low doses of purified autologous total IgG (8 intramuscular injections of autologous total IgG 50 mg for 4 weeks) produced potent immunomodulatory effects (increased serum levels of IL-10 and IFN- γ) and long-term clinical improvement compared to intravenous administration of a high dose of heterologous IgG (1~2 g/Kg of body weight per a patient) in adolescent and adult patients with AD (66, 93). These conflicting results from clinical trials on the intravenous administration of heterologous IgG and intramuscular administration of autologous IgG in patients with AD suggest that differences in the origin of polyvalent IgG (heterologous vs. autologous) and route of administration of polyvalent IgG (intravenous vs. intramuscular) can result in different immunomodulatory effects and clinical efficacy (66).

If polyvalent IgG is used as an “antibody” to neutralize pathogenic antibodies (IgG autoantibodies or IgE antibodies), intravenous administration of polyvalent IgG can be the preferred route of administration (66). However, if polyvalent IgG is used as an “antigen” to activate anti-idiotypic Treg cells in patients with allergic and autoimmune diseases, the intramuscular or subcutaneous administration route might be an ideal route of administration. A specific antigen administered through intravenous injection preferentially interacts with mononuclear cells in the peripheral blood, and a specific antigen administered through intramuscular or subcutaneous injection preferentially interacts with DCs (66).

Intramuscularly administered autologous polyvalent IgG may more efficiently activate pre-existing idiotype-specific Treg cells than with intramuscularly administered heterologous IgG because the pre-existence of idiotype-specific T cell to autologous IgG is an essential prerequisite of the “idiotype-network theory.” The potency of activation of Treg cells induced through intramuscular injection of heterologous polyvalent IgG can be weak because of a smaller number of pre-existing idiotype-specific Treg cells than that in case of intramuscular injection of autologous polyvalent IgG.

Further studies are necessary to evaluate the detailed mechanisms underlying systemic immunomodulation induced by intravenous or intramuscular administration of heterologous or autologous human IgG, especially on the mechanism of anti-idiotypic immunomodulation.

### 3.3 *In vitro* and animal studies about IgG-mediated anti-idiotypic immuno-modulation: lessons from experiments using IgG from donors with various immune backgrounds

#### 3.3.1 Immunomodulatory effects induced by polyvalent IgG from donors with various immune backgrounds

In a mouse model of OVA-allergy, the passive transference of polyclonal IgG from allergen-immunized donors to non-immunized pregnant mice increased IL-10-producing CD4^+^ T cells in the offspring at birth and prevented neonatal OVA-sensitization (47). This effect occurs in the absence of maternal antigen immunization, suggesting that the *in vivo* effect was mediated by idiotypic interactions between the polyvalent IgG from allergen-immunized donors administered to the mother during pregnancy and the fetus lymphocytes (47).

A study suggested that IgG also permeates cells and interacts with intracellular molecules, inhibiting T cell activation after its interaction with nuclear and cytoplasmic components (94). These cell-penetrating antibodies, which could be detected in polyvalent IgG formulations, penetrate various mammalian cell lines and represent ~2% of polyvalent IgG. This possibility also needs to be discussed because it suggests that anti-idiotype IgG may interact with TCRs intracellularly, at an early stage of production of these receptors.

#### 3.3.2 Experimental observations regarding IgG from patients with atopic dermatitis

It has been demonstrated that AD patients can produce detectable levels of anti-IgE autoantibodies in the IgG class (95) and that these antibodies can mediate inflammatory mediators released from basophils and mast cells (96). This observation was confirmed by two studies which showed that the functional activity of IgG anti-IgE autoantibodies from patients with AD induced the release of pro-inflammatory mediators and cytokines from human basophils and mast cells (97, 98).

Human thymic lymphocyte culture with purified IgG showed that pooled IgG from patients with AD could more effectively modulate cytokine production from thymic CD4^+^ T cells than did polyvalent IgG from healthy human subjects (IVIg) (99). This study demonstrates that IgG from patients with AD induced significantly higher production of IL-17 and IL-10 by intrathymic immature double positive and mature CD4^+^ T cells than did polyvalent IgG from healthy individuals (99). In a similar *in vitro* experiment, pooled IgG from patients with AD could induce thymic invariant natural killer T cells (CD1d-restricted T cells that employ an invariant TCR alpha chain and a limited repertoire of beta chains) to produce higher levels of intracellular IL-4, IL-10, and IL-17 than did polyvalent IgG from healthy non-atopic individuals (100). The IgG-reactivity toward 1152 human protein fragments was evaluated in 80 individuals (AD patients and healthy controls), and a significant differential IgG-reactivity to four antigens representing keratin-associated protein 17-1 (KRTAP17-1), heat shock protein family A member 4 (HSPA4), S100 calcium-binding proteins A12, and Z (S100A12 and S100Z) was detected (101). The reactivity to these four antigens was more frequent in the patients with severe AD (66%) than in patients with moderate AD (41%) and healthy controls (25%) (101). These observations suggest that purified IgG from patients with AD can modulate T cells more efficiently than does polyvalent IgG from healthy human subjects (IVIg) (99, 100, 102).

## 4 Six questions on the mechanism underlying polyvalent IgG-induced activation of Treg cells and its clinical application for the treatment of immune diseases

We asked six critical questions regarding the mechanism and its clinical application of polyvalent IgG-induced immune modulation and activation of Treg cells (**Table 1**). We propose one hypothesis to provide an answer to these six questions regarding the mechanism of polyvalent IgG induced activation of Treg cells and its clinical application (**Box 1**). These six questions and one hypothesis should be validated in future studies.

In this hypothesis, subcutaneous or intramuscular injection of autologous polyvalent IgG can induce activation of anti-idiotypic Treg cells to downregulate the production of pathogenic antibodies and exaggerated T cell responses harmful to the host and induce an immune tolerance state and ultimately induce a long-term clinical remission in patients with autoimmune and allergic diseases (**Box 1**). Through the perspective of the idiotypic network theory, immunization with autologous polyvalent IgG might provide a more potent immunomodulatory effects than that provided by heterologous polyvalent IgG in the treatment of autoimmune and allergic diseases.

Box 1 **A new concept of active anti-idiotypic therapy with autologous polyvalent IgG for allergic and autoimmune diseases.**

“Subcutaneous or intramuscular administration of autologous polyvalent IgG can activate anti-idiotypic regulatory T cells that can specifically recognize immunogenic peptides generated from idiotypes of autologous IgG by dendritic cells. The activated anti-idiotypic regulatory T cells can induce an immune tolerance state (recovery of immune homeostasis) and a long-term treatment-free clinical remission in patients with allergic and autoimmune diseases.”

IgG, immunoglobulin G.

## 5 Future research directions (research topics) involving the mechanism and clinical application of polyvalent IgG-induced activation of Treg cells

Polyvalent IgG can induce activation of Treg cells to produce IL-10 and exert a systemic immunomodulatory effect and clinical efficacy in patients with allergic and autoimmune diseases. However, this review also exposed a lack of current knowledge regarding the mechanism of polyvalent IgG-induced immunomodulation including Treg cell activation and its role in the development and maintenance of immune tolerance. Therefore, we provide our hypotheses (unproved speculations on the detailed mechanism) to promote further research on this important but neglected research topic by other colleagues. For future investigations, we propose research topics to answer questions on the detailed mechanism and clinical application of polyvalent IgG-induced immunomodulation (**Table 4**).

## 6 Conclusion

Experimental studies and clinical trials involving Treg cells suggest that polyvalent IgG acts as a stimulator of Treg cells. However, the mechanism underlying the activation of Treg cells by polyvalent IgG is not defined yet. We hypothesize that anti-idiotypic Treg cells can be activated via intramuscular or subcutaneous injection of autologous polyvalent IgG because idiotypes of polyvalent IgG can act as antigens. The activated anti-idiotypic Treg cells secrete IL-10, and IL-10 suppresses Th2 cell responses to allergens and autoimmune T cell responses to self-antigens and thus can induce a long-term clinical remission of allergic and autoimmune diseases. Further studies are needed to evaluate the detailed molecular mechanism underlying polyvalent IgG-induced Treg cell activation and the clinical usefulness of this immunomodulatory strategy for the treatment of allergic and autoimmune diseases.

## Author contributions

All authors contributed in writing and revising the manuscript. DN: Conceptualization, Funding acquisition, Project administration, Supervision, Writing – original draft, Writing – review & editing. JV: Funding acquisition, Writing – original draft, Writing – review & editing. All authors contributed to the article and approved the submitted version.
